# Use of ^13^C-qNMR Spectroscopy for the Analysis of Non-Psychoactive Cannabinoids in Fibre-Type *Cannabis sativa* L. (Hemp)

**DOI:** 10.3390/molecules24061138

**Published:** 2019-03-22

**Authors:** Lucia Marchetti, Virginia Brighenti, Maria Cecilia Rossi, Johanna Sperlea, Federica Pellati, Davide Bertelli

**Affiliations:** 1 Department of Life Sciences, University of Modena and Reggio Emilia, Via G. Campi 103, 41125 Modena, Italy; lucia.marchetti@unimore.it (L.M.); virginia.brighenti@unimore.it (V.B.); johanna.sperlea@ernaehrung.uni-giessen.de (J.S.); davide.bertelli@unimore.it (D.B.); 2 Doctorate School in Clinical and Experimental Medicine (CEM), University of Modena and Reggio Emilia, 41125 Modena, Italy; 3 Centro Interdipartimentale Grandi Strumenti, University of Modena and Reggio Emilia, Via G. Campi 213/A, 41125 Modena, Italy; mariacecilia.rossi@unimore.it; 4 Faculty of Agricultural Sciences, Nutritional Sciences, and Environmental Management, Justus-Liebig University of Giessen, Goethestrasse 58, 35390 Giessen, Germany

**Keywords:** ^13^C-qNMR, HPLC, *Cannabis sativa* L., hemp, cannabinoids, cannabidiol, cannabigerol

## Abstract

*Cannabis sativa* L. is a dioecious plant belonging to the Cannabaceae family. The discovery of the presence of many biologically-active metabolites (cannabinoids) in fibre-type *Cannabis* (hemp) has recently given rise to the valorisation of this variety. In this context, the present study was aimed at the multi-component analysis and determination of the main non-psychoactive cannabinoids (cannabidiol, cannabidiolic acid, cannabigerol and cannabigerolic acid) in female inflorescences of different hemp varieties by means of ^13^C quantitative nuclear magnetic resonance spectroscopy (qNMR). The method proposed here for the first time for the determination of cannabinoids provided reliable results in a competitive time with respect to the more consolidated HPLC technique. In fact, it gave sufficiently precise and sensitive results, with LOQ values lower than 750 μg/mL, which is easily achievable with concentrated extracts, without affecting the quality of ^13^C-qNMR spectra. In conclusion, this method can be considered as a promising and appropriate tool for the comprehensive chemical analysis of bioactive cannabinoids in hemp and other derived products in order to ensure their quality, efficacy and safety.

## 1. Introduction

*Cannabis sativa* L. was one of the first cultivated plants, and it was further adapted by humans to a variety of needs: technical (textiles, construction and paper industries), nutritional (food), medicinal and therapeutic, since it contains over 150 bioactive phytocannabinoids [1]. It is an annual dioecious plant belonging to the Cannabaceae family and it growths almost in every developed country of the world [2]. Taxonomic studies have raised a critical issue, owing to the huge variability of specimens within the same genus but, in the scientific community, a monotypic classification is currently preferred [3]. According to this agreement, one single species (*C. sativa*) has been supposed, and several chemotypes based on the specific cannabinoid profile have been identified [4]. The two principal phenotypes of interest are the drug-type and the fibre-type *Cannabis*, also known as “hemp”. The first is rich in psychoactive Δ^9^-tetrahydrocannabinol (Δ^9^-THC), and it is used for medicinal or recreational purposes, while the second has a content of Δ^9^-THC below 0.2–0.3% in dry weight plant material of the upper one-third of the crop; this legal limit was established by the European Industrial Hemp Association (EIHA), in accordance with international regulations [5,6]. Discovering the presence of many biologically active metabolites also in fibre-type *Cannabis* has allowed the valorisation of this latter variety [7,8]. Moreover, the current favourable regulatory framework encourages pharmaceutical and nutraceutical companies to develop and produce dietary supplements containing plant extracts aimed at the maintenance or the recovery of human and animal wellbeing.

Hemp non-psychoactive cannabinoids include cannabidiol (CBD), cannabigerol (CBG) and their related acids, that accumulate principally within glandular trichomes of female inflorescences [9,10]. Cannabidiolic acid (CBDA) and cannabigerolic acid (CBGA) are biosynthesized in plants as prenylated aromatic carboxylic acids and their neutral counterparts (CBD and CBG) are generated as a consequence of the spontaneous decarboxylation that can occur either by heat or light for instance during long storage processes [7,9,11]. This assumption is supported by the fact that almost no neutral cannabinoid can be detected in the fresh plant material [12]. 

While Δ^9^-THC has led to the discovery of the endocannabinoid system, the molecular basis of CBD activity is still challenging pharmacologists, especially when conventional treatment is of no avail, e.g. for genetic epilepsy [1]. Many studies in the literature have proved that CBD has anxiolytic and neuroprotective properties [13,14,15] and, moreover, that it has anticholinesterase and antiemetic effects [16]. CBD and CBG have both revealed to exert antibacterial properties against a variety of methicillin-resistant *Staphylococcus aureus* strains [17]. CBG has also shown anti-inflammatory activity [18], and it has been associated with muscle relaxation and analgesia by activating *α*-2 receptors [19]. On the other hand, CBDA has been shown to possess an anti-proliferative effect on breast cancer cell migration [20].

Concerning *C. sativa* chemical composition, in addition to the cannabinoid fraction, over 20 flavonoids have been identified and they belong to the chemical classes of flavones or flavonols. In particular, cannflavin A, cannflavin B and cannflavin C are the major hemp prenylated flavanones [7], that are peculiar for this plant. Hemp seed-oil has demonstrated antioxidant activity, which is probably due to the high content of flavonoids [21,22]. When utilized as a food source, hemp seeds have exhibited excellent nutraceutical properties [23]. The seed oil is rich in phytosterols, vitamins, minerals and amino acids; in particular, it has a high content of essential polyunsaturated fatty acids, such as linoleic acid (18:2, ω-6) and α-linolenic acid (18:3, ω-3) [23]. *Cannabis* essential oil extracted from the inflorescences is composed of monoterpenes (β-myrcene, α- and β-pinene and limonene) and sesquiterpenes (β-caryophyllene, α-humulene and caryophyllene oxide) [8], which exert synergistic actions against cancer and they showed analgesic properties [24]. Hemp essential oil has today different applications in nutraceuticals, cosmetics and insect control purposes in agriculture [25]. 

In addition to separation techniques involving chromatography, Nuclear Magnetic Resonance (NMR) spectroscopy can be applied to the analysis of the composition of plant extracts, in the structure elucidation of compounds in metabolomics as well as in their quantification [26]. The measuring principle is based on the effect of an external magnetic field on spin possessing atomic nuclei ^1^H and ^13^C. Due to changes in the intramolecular magnetic field around an atom, absorption and emission of electromagnetic radiation take place. Then, the resonance is detected with a radio receiver. The Fourier transform converts the complex time domain signal emitted by the nuclei into the frequency domain spectrum. 

Quantitative NMR (qNMR) refers to the application of NMR in order to determine the concentration of one or more target compounds in solution. The underlying principle is the direct proportional correlation of the area of a signal to the number of nuclides contributing to it. This proportionality depends on pulse excitation, delay time and broad-band decoupling; therefore, constant conditions are necessary [26,27]. Nowadays, there is an increasing interest in the application of qNMR, due to its numerous advantages over chromatographic methods. Indeed, qNMR enables the simultaneous identification and quantification of many analytes, also chemically very different, in complex mixtures with a reduced time of analysis and a lower solvent usage, compared with the HPLC technique [28,29].

In the light of all the above, the aim of this study was the development of a reliable ^13^C-qNMR method for the comprehensive characterisation and determination of the main non-psychoactive cannabinoids (CBD, CBDA, CBG, CBGA) in eight different hemp varieties. The obtained results were compared with those provided by a previously developed method based on high-performance liquid chromatography (HPLC) [7,8,30]. The ^13^C-qNMR method developed in this study for the first time produced satisfactory results in a competitive time with respect to the most widespread and consolidated technique for cannabinoid analysis (HPLC), thus demonstrating to be suitable for a multi-component analysis of hemp extracts. This technique could also be useful to gain a holistic view of the composition and chemical profile of different hemp varieties, and it can be applied for the quality control of both the plant material and derivatives.

## 2. Results and Discussion

### 2.1. NMR Spectroscopic Data of Extracts from Hemp Inflorescences

**Figure 1:**
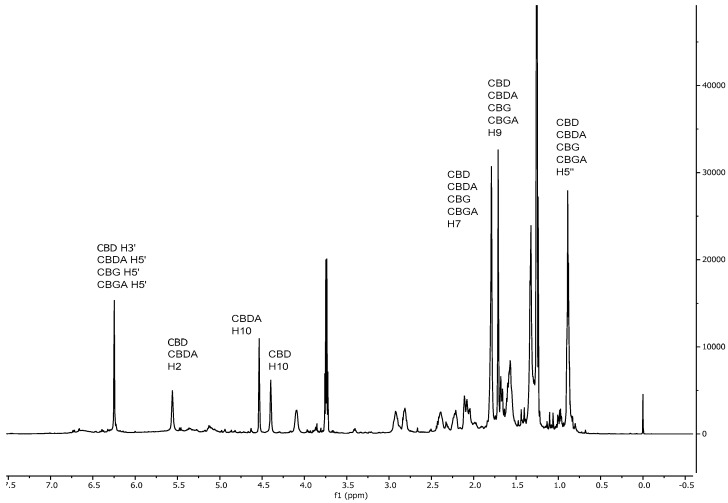
Typical ^1^H spectrum of a hemp ethanolic extract.

**Figure 2:**
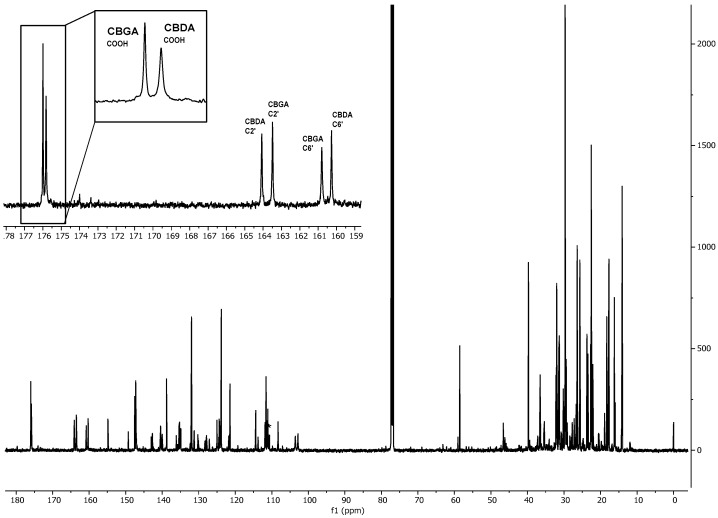
Typical ^13^C spectrum of a hemp ethanolic extract; the enlarged region shows the high spectral resolution.

**Figure 3:**
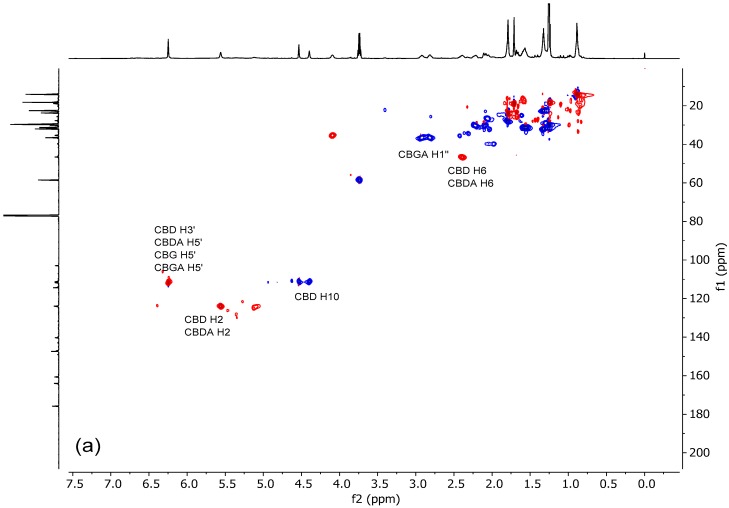

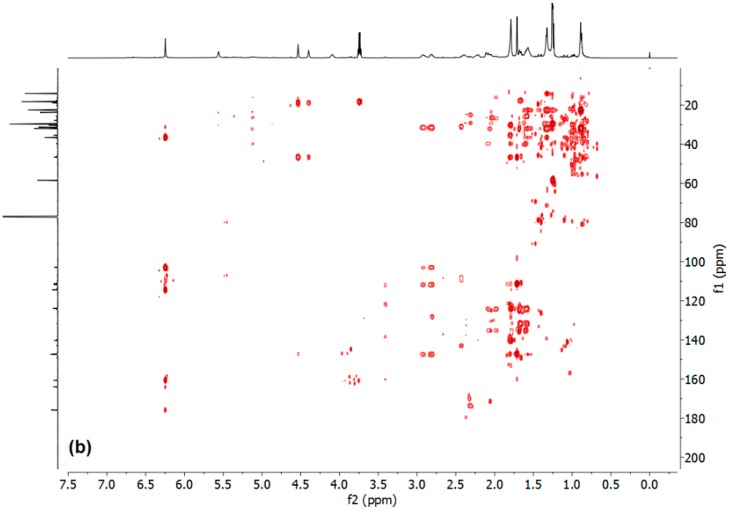
Typical HSQC (**a**) and HMBC (**b**) spectra of a hemp ethanolic extract.

Typical ^1^H, ^13^C, HSQC and HMBC spectra of a Santhica extract are shown in Figure 1, Figure 2 and Figure 3.

**Figure 4:**
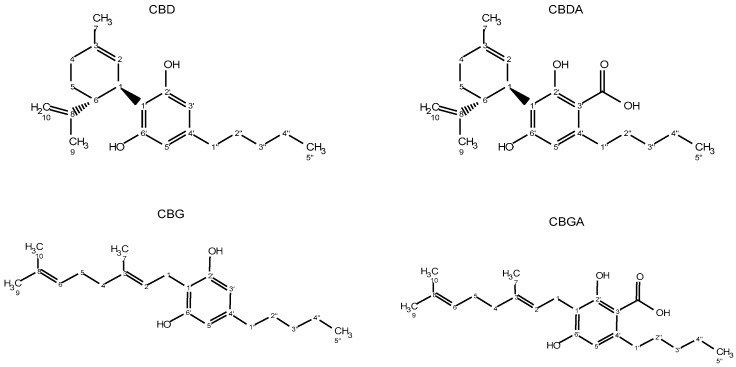
Chemical structures of hemp non-psychoactive cannabinoids.

The spectra of samples and standard compounds were compared, and the results led to the assignments for CBD, CBG, CBDA and CBGA shown in Figure 4. The chemical shifts in deuterated chloroform for ^1^H and ^13^C are shown in Table 1 and Table 2, expressed as ppm related to tetramethylsilane (TMS). 

As shown in Figure 1, the protonic spectrum presents a huge number of overlapped peaks, thus requiring an extensive use of deconvolution for signal integration, which can introduce some accuracy issues. To overcome this problem, in this work, ^13^C-qNMR was evaluated for the first time for the determination of cannabinoids in hemp extracts [26]. As shown in Figure 2 and Table 2**,** carbon signals are better resolved and easier to deal with for quantitative purpose. In this context, in these experimental conditions evident signal doubling, due to conformational equilibria, and line broadening did not occur. In particular, all the signals used for CBD and CBG quantification showed a measured average half width of 1.25 Hz (maximum 1.5 Hz). CBDA and CBGA signals were slightly broader; in this case, the average half width was 3.3 Hz (maximum 4 Hz). This line broadening effect did not invalidate or affect the quantification of these compounds, considering that the differences between qNMR and HPLC data are limited and not significant (Table 3).

On the other hand, it is well known that ^13^C-qNMR presents some complexities, which often makes the use of this technique quite problematic. In particular, the major issues related to ^13^C-qNMR are:

1. low sensitivity, due to the lower ^13^C isotopic abundance (1%) in comparison to ^1^H;
2. carbons typically have long T_1_ relaxation time; in this way, signals could not reach their full intensity;
3. the ^13^C signal intensity is affected by the Nuclear Overhauser Enhancement (NOE) effect, that depends on ^13^C type (from primary to quaternary).

In order to reduce the impact of these issues, the following measures were applied. With regards to the sensitivity problem, it was possible to acquire spectra of concentrated hemp extracts without disregarding the spectrum quality in terms of resolution, intensity and resonance shifting, due to the matrix effect. To overcome the issue related to ^13^C long relaxation time, T_1_ for each carbon of standard compounds was tentatively measured. Some of the nuclei did not provide results in an acceptable time, due to the molecule structure or to the low amount of standard available (CBDA and CBGA). For this reason, only signals with T_1_ < 2 s were initially selected for quantification (marked with * in Table 2). Furthermore, some characteristic signals with T1 > 2 s were also tentatively used for the two acidic compounds (marked with ** in Table 2). Finally, a pulse delay (D_1_) equal to 10 s (5*T_1_) was used. This parameter allowed us to obtain the full relaxation in an acceptable time, thus making possible to perform the analysis in a competitive time (47 min) with respect to the most common HPLC technique. The last question was solved by lowering to zero the proton decoupling power. 

### 2.2. ^13^C-qNMR Method Validation

The Concentration Conversion Factor (CCF), obtained from the analysis of standards and used by the software to calculate the concentration of compounds in samples, was measured in the range of expected concentrations, by diluting a CBD standard solution. The CCF value was constant (0.71605 ± 0.00004) in all the considered concentration ranges, thus assuring a good linearity (*r*^2^ = 0.9998). The method proposed here provided also a good intra- and inter-day precision, with percentage relative standard deviation (%RSD) values of 0.8 and 1.5%, respectively. With regards to the limit of detection (LOD), in these experimental conditions the lowest concentrations which presented clearly distinguishable signals from the spectral background were 0.6, 0.5, 0.6 and 0.5 mM for CBD, CBDA, CBG and CBGA, respectively, corresponding to LOD values of 188, 180, 190 and 180 μg/mL in the NMR tube. The lowest quantification limits (LOQ), by applying the below mentioned S/N restriction, corresponded to 2.11, 2.15, 2.10 and 2.18 mM for CBD, CBDA, CBG and CBGA, respectively, with relative LOQ values of 660, 746, 664 and 746 μg/mL in the NMR tube.

### 2.3. Quantitative Analysis of Hemp Inflorescences

Quantitative results of hemp inflorescences obtained by ^13^C-qNMR are shown in Table 3, compared to those obtained by HPLC [7,8,30]. Data are expressed as mean (mg/g) ± standard deviation (SD), dry weight. These values correspond to the overall means of three replicates for all the considered signals for each compound. The results provided by ^13^C-qNMR and HPLC are comparable, without meaningful differences. It should also be considered that the signals tentatively used for CBDA and CBGA with T_1_ > 2 s (** in Table 2) provided very similar quantitative results as well as those with the lower relaxing times, thus demonstrating the method’s ruggedness. In particular, ^13^C-qNMR seems to slightly underestimate the CBD and CBG content with respect to HPLC, since this situation occurs for every cultivar. The mean recovered amounts of CBD and CBDA were significantly higher respect those of CBG and CBGA; this finding is supported by other studies, in which the measured concentrations for these cannabinoids were undetectable or very low [8,30]. In the majority of samples (Antal, Carmagnola, China, Fibrante, Futura and Santhica), the acidic compounds were more concentrated with respect to their neutral counterparts, demonstrating that the extraction method is able to limit the decarboxylation process. Codimono was the cultivar which provided the highest content of CBD, while Futura was the one with the highest amount of CBDA. With regards to CBG, only one hemp variety (Santhica) showed a concentration over the LOQ value. CBGA contents were determined instead for Antal, Carmagnola, Futura and Santhica, with the latter two varieties being the richest ones.

The ^13^C-qNMR method here proposed for the quantification of non-psychoactive cannabinoids in hemp provided reliable results in comparison to the most commonly and consolidated HPLC technique. The validation confirmed the ruggedness of this qNMR approach, which was found to be precise and sufficiently sensitive. In conclusion, this ^13^C-qNMR method is suitable and advantageous for a multi-component analysis of cannabinoids in hemp extracts. This technique could be useful to gain a holistic view of the composition and chemical profile of different hemp varieties, and it could be applied in the quality control of both the plant material and its derivatives.

## 3. Materials and Methods 

### 3.1. Chemicals and Solvents

CBD, CBDA, CBG and CBGA standard solutions (1 mg/mL in methanol or acetonitrile) were purchased from Cerilliant (Round Rock, TX, USA). Deuterated chloroform (CDCl_3_), tetramethylsilane (TMS) and ethanol (EtOH) of analytical grade were from Sigma-Aldrich (Milan, Italy). 

### 3.2. Hemp Plant Material

Eight samples of fibre-type female inflorescences were analysed in this study, including Antal, Carma, Carmagnola, China, Codimono, Fibrante, Futura and Santhica. These samples (about 100–500 g of dry material each), belonging to different breeding lines, were cultivated under the same agronomic conditions and they were kindly provided by Dr. Gianpaolo Grassi of the research centre CREA-CIN (Rovigo, Italy). Each sample was certified for a content of Δ^9^-THC below 0.2% (*w/w*). All the samples considered in this study were approved for commercial use by the European Union [31].

### 3.3. Sample Preparation

Hemp inflorescences were manually separated from twigs and seeds. After this procedure, the samples were stored at +4.0 °C until analysis. Cannabinoids were extracted from hemp inflorescences by means of dynamic maceration, as previously described in detail [7,8]. A weighed amount of each sample (1 g) was extracted with 20 mL of absolute EtOH at room temperature for 15 min, under magnetic stirring. The solution was then paper filtered and the residue was extracted with the same procedure twice more with 20 and 10 mL of same solvent, respectively. The filtrates were then combined and adjusted to 50 mL with EtOH in a volumetric flask. The extraction procedure was repeated twice for each sample. 

To prepare NMR tubes, the extracts were evaporated under vacuum at 30 °C and the residue was dissolved in 1 mL of CDCl_3_ (0.03% TMS); finally, 600 μL of this solution were transferred to a WILMAD^®^ NMR tube, 5 mm, Ultra-Imperial grade, 7 in. L, 528-PP (Sigma-Aldrich, Milan, Italy). 

### 3.4. NMR Spectroscopy and Spectra Acquisition Procedures

To characterize samples ^1^H-NMR, ^13^C-NMR, two-dimensional ^1^H-^13^C Heteronuclear Multiple-Bond Correlation (HMBC) and ^1^H-^13^C Heteronuclear Single Quantum Coherence (HSQC) spectra were acquired with a Bruker FT-NMR Avance III HD 600 MHz spectrometer (Bruker Biospin GmbH Rheinstetten, Karlsruhe, Germany). All the experiments were performed at 300 K and non-spinning. The assignments for the major compounds were carried out by using standard compounds and by comparison of ^1^H-NMR and ^13^C-NMR spectra with literature data.

^1^H-NMR (i.e. 1D NOESY) experiments were acquired by using the Bruker sequence “noesygppr1d” for residual CDCl_3_ pre-saturation; the acquisition parameters were as follows: time domain (number of data points), 64 K; dummy scans, 0; number of scans, 128; acquisition time, 3.41 s; delay time, 8 s; spectral width, 16 ppm (9615 Hz), fid resolution 0.2935 Hz; digitization mode, baseopt. Total acquisition time was 24 min and 26 s. 

^13^C-qNMR quantification experiments were performed by using a 1D inverse gated decoupling sequence to avoid NOE during relaxation (Bruker sequence: “zgpg_pisp_f2.fas”). The acquisition parameters were as follows: time domain (number of data points), 64 K; dummy scans, 10; number of scans, 256; acquisition time, 1 s; delay time, 10 s; spectral width, 220 ppm (33333 Hz); fid resolution, 1.017 Hz; digitization mode, baseopt; the proton decoupling power, 0 db. Total acquisition time was 47 min. 

The acquisition parameters for the HMBC experiments (Bruker sequence “hmbcetgpl3nd” with three-fold low-pass J-filter to suppress one-bond-correlations) were as follows: time domain, 4 K in the acquisition or direct HMBC dimension F2 (^1^H) and 256 in indirect HMBC dimension F1 (^13^C); dummy scans, 16; number of scans, 8; acquisition time, 0.310 s; delay time, 1.5 s; delay time for evolution of long range couplings, 62.5 μs; spectral width, 11 ppm (6602 Hz) in F2 (^1^H) and 210 ppm (31692 Hz in F1) (^13^C); fid resolution, 3.22 Hz (F2) 247.60 Hz (F1); digitization mode, baseopt. J-coupling for one-bond correlations suppression: 1J (XH) Min 120 Hz, 1J (XH) Max 170 Hz; J (XH) long range, 8 Hz. Total acquisition time was 1 h and 5 min. 

The acquisition parameters for the HSQC experiments (Bruker sequence “hsqcedetgpsp.3”) were as follows: time domain, 2 K in the acquisition or direct HSQC dimension F2 (^1^H) and 256 in indirect HSQC dimension F1 (^13^C); dummy scans, 16; number of scans, 8; acquisition time, 0.156 s, delay time, 2 s; spectral width, 11 ppm (6602 Hz) in F2 (^1^H) and 210 ppm (31693 Hz in F1) (^13^C); fid resolution, 6.45 Hz (F2) 247.60 Hz (F1); digitization mode, baseopt. J-coupling for one-bond correlation selection 1J (XH) 145 Hz. Total acquisition time was 1 h and 15 min. 

After introducing the sample into the probe, at least 5 min were required to achieve a thermal equilibrium. Afterwards, the magnetic field was locked, the probe head was tuned and matched and, finally, the sample was shimmed. To assure the highest reproducibility, all these procedures were automatically executed. The correct 90° pulse was calibrated for protonic and bi-dimensional spectra with the Bruker AU program “pulsecal” and the receiver gain was set.

### 3.5. ^13^C-qNMR Procedure

Quantitative NMR was performed by using the Concentration Conversion Factor (CCF) method, implemented in Mnova® 12.0 (Mestrelab Research, S.L., Santiago de Compostela, Spain) software. A CBD standard solution (1 mg/mL) was used as the external reference compound. The CCF provided by this analysis was verified and corrected by using a commercial standard of 40% dioxane in benzene-*d_6_* (Bruker Biospin GmbH Rheinstetten, Karlsruhe, Germany). This solution is normally used for ^13^C sensitivity test; in our case, the signal at 66.69 ppm (corresponding to the four equivalent carbons) was used for the quantification. In order to determine the correct delay time (D_1_), T_1_ was measured for both dioxane and the standard compounds, when possible. In the case of acidic compounds, the T_1_ of the signals related to the presence of carboxylic groups was directly estimated in the sample richest in CBDA and CBGA. For T_1_ determination, the Bruker Sequence “T_1_IR” was employed. The following parameters were applied: a list of 8 increasing delay times (from 0.2 to 20 s); delay time, 20 s; number of scans, 32; total acquisition time, 2 h. A D_1_ time equal to 10 s (5*T_1_) was set to ensure the nuclei complete relaxation during acquisition. Moreover, only signals presenting a signal to noise ratio (S/N) ≥ 50 were used for quantification. An exponential window function, with a line broadening (lb) equal to 0.3, was selected for spectra transformation. Each spectrum was calibrated according to TMS signal and then an automatic zero order phase and base line correction were applied, before peak integration. The quantification tool of Mnova requires a multiplet analysis for the integration; therefore, after the initial spectra processing, a manual multiplet analysis was performed and the peak area of signals belonging to the target compounds were compared to the area of those generated by the CBD standard solution, whose spectrum was acquired under the same conditions. The results were then corrected with the correction factor obtained from the dioxane standard solution. The resonances suitable for quantification are marked with * in Table 1. In general, more than one signal should be integrated for each compound. In the cases of CBD and CBDA, eight carbons having the required characteristics were chosen and they were used to calculate the average concentration of related compounds. For CBG and CBGA, six resonances were selected. In addition, both CBDA and CBGA showed three well resolved peaks, in the region ranging from 160 to 180 ppm, related to the presence of the carboxylic group on the aromatic ring, which were also used for quantification and they are marked with ** in Table 1. All the acquisitions were performed in triplicate.

### 3.6. HPLC Analysis

The HPLC analysis was intended to verify ^13^C-qNMR results and it was carried out by using the previously validated method [7,8,30].

### 3.7. Method Validation

Since that the validation procedure for the extraction and HPLC analysis of hemp extracts has already been discussed [7,8,30], the attention was focused on the ^13^C-qNMR method in the present work. Linearity was investigated by analyzing both standard solutions at different concentrations (from 2 to 100 mM) and a sample prepared with different dilution factors. Linearity was evaluated by calculating the CCF of the selected signals for quantitative analysis in the aforementioned samples and verifying its constancy inside the concentration range. Precision was assessed by analyzing the same extract five times for three different days. The LOD for the target compounds (CBD, CBDA, CBG and CBGA) was determined by gradually diluting a sample containing measurable amounts of the four molecules, until a S/N ratio equal to 3 was reached. Since only signals with a S/N ratio > 50 were considered to be suitable for quantification, the LOQ value was calculated by considering the area of a signal with a S/N equal to 49.

## Figures and Tables

**Table 1:** ^1^H assignments for hemp cannabinoids in CDCl_3._

	CBD	CBDA	CBG	CBGA
	^1^H ^a^	^1^H ^a^	^1^H ^a^	^1^H ^a^
1	3.86 (1H, m, 11.8 Hz)	3.88 (1H, m, 11.0 Hz)	1.33 (2H, d, 7.0 Hz)	1.79 (2H, d, 7.4 Hz)
2	5.55 (1H, s)	5.55 (1H, s)	5.27 (1H, t, 7.0 Hz)	5.27 (1H, t, 7.0 Hz)
3	-	-	-	-
4	2.10 (1H, m); 2.20 (1H, m)	2.10 (1H, m) 2.20 (1H, m)	2.04 (2H, t, 6.6 Hz)	2.04 (2H, t, 6.6 Hz)
5	1.84 (2H, q, 3.0 Hz)	1.86 (2H, q, 3.0 Hz)	2.07 (2H, q, 6.5 Hz)	2.07 (2H, q, 6.5 Hz)
6	2.40 (1H, m)	2.40 (1H, m)	5.56 (1H, m)	5.05 (1H, t, 6.6 Hz)
7	1.79 (3H, s)	1.79 (3H, s)	1.79 (3H, s)	1.80 (3H, s)
8	-	-	-	-
9	1.66 (3H, s)	1.72 (3H, s)	1.68 (1H, s)	1.67 (3H, s)
10	4.40 (2H, m)	4.54 (2H, m)	1.58 (1H, s)	1.58 (3H, s)
1’	-	-	-	-
2’	-	-	-	-
3’	6.26 (1H, brs)	-	6.0 (1H, s)	-
4’	-	-	-	-
5’	6.16 (1H, brs)	6.26 (1H, s)	6.24 (1H, s)	6.23 (1H, s)
6’	-	-	-	-
1’’	2.42 (2H, t, 7.5 Hz)	2.42 (2H, t, 7.5 Hz)	2.44 (2H, t, 7.5 Hz)	2.88 (2H, t, 7.6 Hz)
2’’	1.57 (2H, m)	1.58 (2H, m)	1.54 (2H, m)	2.10 (2H, m)
3’’	1.30 (2H, m)	1.33 (2H, m)	1.56 (2H, m)	1.32 (2H, m)
4’’	1.31 (2H, m)	1.34 (2H, m)	1.57 (2H, m)	1.32 (2H, m)
5’’	0.89 (3H, t, 6.8 Hz)	0.90 (3H, t, 6.8 Hz)	0.88 (3H, t, 6.9 Hz)	0.89 (3H, t, 6.9 Hz)

^a^ The number of protons, multiplicity and coupling costants are shown in brackets.

**Table 2:** ^13^C assignments for hemp cannabinoids in CDCl_3._

	CBD		CBDA		CBG		CBGA	
	^13^C	T_1_ s	^13^C	T_1_ s	^13^C	T_1_ s	^13^C	T_1_ s
1	37.0 *	1.32	36.7 *	1.51	22.5 *	1.35	25.7 *	1.54
2	124.3 *	1.20	124.0 *	1.35	121.8 *	1.89	121.5 *	1.92
3	139.9	5.52	140.3	-	140.2	6.02	138.8	-
4	31.5	2.10	31.3	-	39.7	3.00	39.7	-
5	28.4	0.97	27.8	-	26.3	1.02	26.4	-
6	46.2 *	1.90	46.6 *	1.91	123.8	2.05	123.8	-
7	23.4 *	1.41	23.7 *	1.61	16.0 *	1.23	16.2 *	1.40
8	149.9	5.52	147.2	-	132.0	5.50	131.7	-
9	20.3	2.67	18.9	-	23.4	2.68	23.4	-
10	110.8 *	0.96	111.3 *	1.10	17.6	1.03	17.8	-
1’	113.8	8.08	114.4	-	110.7	6.66	111.1	-
2’	156.0	-	164.1 **	2.5	154.9	-	163.5 **	2.65
3’	108.3	-	103.1	-	108.3	-	103.6	-
4’	142.9	-	147.2	-	142.7	-	147.5	-
5’	108.3	-	111.7	-	108.3	-	111.8	-
6’	153.9	-	160.1 **	2.7	154.9	-	160.8 **	2.81
1’’	35.5 *	1.95	35.4 *	1.95	35.6 *	1.23	36.3 *	1.25
2’’	30.4 *	1.12	29.7 *	1.12	30.8 *	1.06	30.2 *	1.10
3’’	30.7 *	1.47	30.2 *	1.47	31.5 *	1.50	31.4 *	1.52
4’’	22.5	3.82	22.5	3.88	22.5	3.90	22.5	3.92
5’’	14.1	3.19	14.1	3.23	14.1	3.20	14.1	3.36
-COOH	-	-	175.3 **	3.1	-	-	176.0 **	3.11

* Signals with T_1_ < 2 s used for quantification. ** Signal with T_1_ > 2 s used for quantification. T_1_ was measured, when possible, by using standard compounds.

**Table 3:** qNMR data (mg/g) of cannabinoids in hemp inflorescences compared with HPLC.

	CBD		CBDA		CBG		CBGA	
	qNMR	HPLC *	qNMR	HPLC *	qNMR	HPLC	qNMR	HPLC
**Antal**	2.2 ± 0.2	2.4 ± 0.1	15.7 ± 0.5	15.5 ± 0.7	<LOQ ^b^	0.3 ^a^	1.2 ± 0.2	0.6 ^a^
**Carma**	5.8 ± 0.4	6.0 ± 0.3	<LOQ^b^	2.2 ± 0.1	<LOQ ^b^	0.5 ± 0.1	<LOQ ^b^	0.4 ^a^
**Carmagnola**	3.0 ± 0.4	3.3 ± 0.3	17.3 ± 0.6	16.7 ± 1.2	<LOQ ^b^	0.1 ^a^	1.2 ± 0.1	0.7 ± 0.1
**China**	8.0 ± 0.5	8.4 ± 0.1	18.3 ± 0.7	17.2 ± 0.8	<LOQ ^b^	0.2 ± 0.1	<LOQ ^b^	0.5 ± 0.1
**Codimono**	9.3 ± 0.6	9.8 ± 0.3	1.1 ± 0.2	2.9 ± 0.3	<LOQ ^b^	0.1 ^a^	<LOQ ^b^	< LOQ ^d^
**Fibrante**	6.6 ± 0.5	7.9 ± 0.5	14.7 ± 0.3	14.5 ± 1.1	<LOQ ^b^	0.2 ^a^	<LOQ ^b^	0.4 ^a^
**Futura**	2.8 ± 0.2	3.3 ± 0.1	34.0 ± 0.6	33.8 ± 0.3	<LOQ ^b^	<LOQ ^c^	1.7 ± 0.3	1.3 ± 0.1
**Santhica**	2.1 ± 0.1	2.3 ± 0.2	18.7 ± 0.8	17.3 ± 2.4	1.2 ± 0.1	1.4 ^a^	9.4 ± 0.4	9.9 ± 0.2

* HPLC data of CBD and CBDA are reprinted with permission from Protti, M.; Brighenti, V.; Battaglia, M.R.; Anceschi, L.; Pellati, F.; Mercolini, L. Cannabinoids from *Cannabis sativa* L.: a new tool based on HPLC−DAD−MS/MS for a rational use in medicinal chemistry. *ACS Med. Chem. Lett.* in press. Copyright 2019 American Chemical Society. ^a^ SD < 0.05. ^b^ LOQ values are shown in paragraph 2.2. ^c^ CBG LOQ 1.8 μg/mL [7,8]. ^d^ CBGA LOQ 2.5 μg/mL [7,8].
